# Development and psychometric evaluation of the sexual knowledge and attitudes scale for premarital couples (SKAS-PC): An exploratory mixed method study

**Published:** 2018-01

**Authors:** Zohreh Sadat, Fazlollah Ghofranipour, Seyed Ali Azin, Ali Montazeri, Azita Goshtasebi, Azam Bagheri, Elham Barati

**Affiliations:** 1 *Department of Midwifery, Nursing Trauma Research Center, Kashan University of Medical Sciences, Kashan, Iran.*; 2 *Department of Health Education and Health Promotion, Faculty of Medical Sciences, Tarbiat Modares University, Tehran, Iran.*; 3 *Department of Health Promotion, Institute for Health Sciences Research, ACECR, Tehran, Iran.*; 4 *Health Metrics Research Center, Institute for Health Sciences Research, ACECR, Tehran, Iran.*; 5 *Department of Family Health, Institute for Health Sciences Research, ACECR, Tehran, Iran.*; 6 *Department of Midwifery, Kashan University of Medical Sciences, Kashan, Iran.*; 7 *Student Research Committee, Faculty of Medicine, Kashan University of Medical Sciences, Kashan, Iran.*; * Zohreh Sadat and Fazlollah Ghofranipour are co-corresponding authors.

**Keywords:** Sexual health, Knowledge, Attitudes, Psychometrics

## Abstract

**Background:**

Designing a valid and reliable questionnaire that allows a fair evaluation of sexual knowledge and attitudes and develop a proper sexual educational program is necessary.

**Objective:**

The present study was designed to develop and psychometric evaluation of the sexual knowledge and attitudes scale for premarital couples.

**Materials and Methods:**

An exploratory mixed method study was conducted in two phases; in the first, in order to develop a questionnaire an item pool was generated on sexual knowledge and attitudes through focus group discussions and individual interviews. In the second phase, the psychometric properties of the questionnaire were examined. For this purpose, face validity, content validity as well as construct validity were conducted. Reliability was assessed by the Cronbach’s alpha coefficient to assess internal consistency and test-retest reliability.

**Results:**

In the first phase an item pool with 88 questions was generated (sexual knowledge 45 items and sexual attitudes 43 items). In the second phase, the number of final items reduced to 33 and 34 items of sexual knowledge and sexual attitudes respectively, through exploratory factor analysis (EFA). Five factors for sexual knowledge and six factors for sexual attitudes identified by EFA. The Cronbach’s alpha coefficient for two sections was 0.84 and 0.81 respectively. The test- retest correlations for sexual knowledge and sexual attitude was 0.74 and 0.82 respectively.

**Conclusion:**

The findings suggest that the Sexual Knowledge and Attitudes Scale for Premarital Couples is a valid and reliable instrument. Further studies are needed to establish stronger psychometric properties for the questionnaire.

## Introduction

Sexual health is one of the main aspects of healthy living that affects people of all ages (1). Sexual health definition is the state of physical, emotional, psychological, and social Well-being is relationship sexualityr; this is not just a lack of disease (2). Studies have shown that in the Middle East and North Africa, including Iran, sexual issues are very sensitive topics. Moreover, in these countries health care provider often lack sufficient training. In addition, healthcare systems naturally do not offer a coordinated package of sexual health services to people who are planning to marry (3). Thus, in societies such as Iran premarital Couples especially women are in great need of sex education (4). In Iran, historically, a highly strong religious and socio-cultural system has always influenced sexual relationships, postponing all types of sexual contact until marriage and considers any pre-marital sexual contact and intimacy a sin (5, 6). However, the age of marriage such as other societies has risen in Iran (7-9). Contrary to expectation, many youths start sexual relationships very early. A recent study shows that the prevalence of premarital activities among young people is increasing (10). In fact, many young people in Iran do not have information about sex life (5, 11). 

Proper training on sexual issues prevents the incidence of sexual disorders and high-risk behaviors. Moreover, sexual education helps couples to be more sensitive toward their interpersonal relationships, which eventually creates a stronger bond and gives them a greater enjoyment of sex (12-15). In Iran, the ministry of health is responsible for the organization of the premarital counseling programs in all across the country. The purpose of these classes was to raise the level of couples' knowledge regarding different methods of contraception, communication skills, genetic counseling and sexual health, and all couples are required to register for these classes (16). 

However, studies in Iran show that pre-marriage counseling programs do not provide sexual health needs of couples (4, 17, 18). A descriptive study was performed in Tabriz city to evaluate the quality of pre-marriage counseling services in health centers. (19). Results showed more than 50% of participants indicated that sexual education was poor. Another study was conducted to identify challenges of pre-marriage counseling program in Iran (20). The results showed that the need for knowledge and sexual skills of married couples is not considered in the pre-marital training classes. However, the implementation of any sexual health education program requires attention to the needs of couples and the removal of barriers to healthy sexual behaviors for the promotion of knowledge and attitudes about sexual health. (21). Considering the role of sexual knowledge and sexual attitudes on improvement of sexual health, it is important to use the valid and reliable questionnaires for their assessment. 

One of such scales is a questionnaire developed by “Anne Hooper’s” that measured sexual issues in both sexes. This scale has been applied in some studies in Iran (22-24), there are several socio-cultural concerning about the using of the questionnaire and the same scales. Hendrick and colleagues have also designed sexual attitudes questionnaire, it included some dimensions such as nudity, premarital sex, and homosexuality (25). Based on the current culture in Iran, the participants would probably be irritated by these type of questions or do not properly respond. In this regard, validity and reliability an available questionnaire in this filed ʺAcquisition of sexual Information Test" was assessed in Iran, results showed it seems to be culturally inappropriate for Iranian women (26). To address this goal, a localized questionnaire that can assess sexual knowledge and attitudes is necessary. Therefore, recently, some Iranian researchers tried to develop scales for assessing sexual knowledge and attitude adopted with the socio-cultural context of Iran (27-29). 

However, most of them are not tailored to the needs target group or specific issues of sexual knowledge and attitudes were unconsidered. Moreover, studies conducted in Iran to determine women's sexual knowledge and attitudes are quantitative. Yet, quantitative methods cannot deeply examine participants' perceptions and attitudes, and real experiences should be taken into account (30). Furthermore, use of textual and visual documents is necessary for determine couple's knowledge and attitudes of sexual health issues (31). 

The purpose of the present study was to develop and assess the psychometric properties of a newly developed questionnaire in order to identify sexual knowledge as well as attitudes in premarital couples.

## Materials and methods

The present exploratory mixed method study was conducted in two steps; in the first phase (qualitative phase), the items were generated and the questionnaire was developed and in the second phase (quantitative study), the psychometric properties (validity and reliability) of the questionnaire were assessed.

**Phase 1: Item generation and questionnaire development**

**Methods**

A qualitative process was carried out through focus group discussions (FGDs) and individual interviews from May to April 2014.

**Participants and data collection**

We recruited 66 women and men in two steps containing FGDs and individual interviews using purposive sampling. Eight meetings were held on men and women: In all, four with women (n=29) and four with men (n=31). Participants were recruited from pre-marriage couples attending to a health center for premarital educational classes (n=48) and married women and men (n=12) in Kashan, Iran. Each FGD included 6 to 8 men or women aged 18-43 yr who were volunteers to take part in this study. Maximum diversity in sampling was performed to obtain data saturation (32). Maximum variant was considered in order to select samples from different age, and socio-economic groups. 

The FGDs were facilitated by using a semi-structured that began with the open-ended questions such as what do you know about sexual issues; what do you think about sexual issues? Subsequently, based on the responses received from the participants, further questions were asked. The focus groups lasted 60-80 min. It is worth noting that in the Islamic Republic of Iran all premarital couples have to participate in pre-marriage educational classes. These sessions are only 1 hr in which some topics on reproductive health are orally presented.

Interviews: The individual interviews were conducted on 6 participants (two health care providers who were instructors for the pre-marriage education classes as well as four key informants). Health care providers and key informants were asked about, issues that premarital couples might need to know, and what they thought about sexual issues. Individual interviews lasted 25-45 min. Data saturation was achieved by four individual interviews and six focus groups. 

**Data analysis**

The interviews were audio-recorded to be transcribed and ultimately analyzed. Qualitative content analysis method was conducted based on Elo (33): All interviews included GDs and the individual was transcribed and analyzed prior to the next interview. Comprehensive data was achieved by reading and re-reading by researchers. An initial list of codes developed by research team. The primary categories were achieved by initial codes. The main categories were delivered by combining related categories. Abstraction was performed. Data reliability was achieved through several methods. Participants were guaranteed to keep secret names. The extracted data and codes were assessed by participants and verified. The interviews' text, codes and categories extracted approved by the research team and another people, who experienced in qualitative research and not related to the current study. Some people who did not take part in the research verified the adaptabilityof the results.

**Results**

Finally, an item pool containing 45 items for ‘sexual knowledge’ and 43 items for ‘sexual attitudes were generated for psychometric properties. Three choices were given for each item in sexual knowledge section: true (score=1), false (score=-1) or don’t know (score=0) and each item in sexual attitude section was rated on a five-point Likert scale (completely agree to completely disagree). Higher scores on both sections represent having a higher sexual knowledge and positive sexual attitudes. Each item in two sections was either a positive or a negative statement. Reverse scoring was carried out for negative statements. 

**Phase 2: Psychometric evaluation (validity and reliability) of the sexual knowledge and attitudes scale (SKAS-PC)**

**Participants and data collection**

A cross-sectional study was carried out in order to evaluate psychometric properties of the pre-final version of SKAS-PC. A sample of couples referred to Gholabchi health center in Kashan, Iran between Aprils to August 2014 was recruited through convenience sampling. Gholabchi health care center is the only pre-marriage testing center in Kashan. They were eligible if they were literate, 16-45 yr old, never been previously married and volunteered to participate in the study. As recommended a sample of 340 participants was thought (10 individuals per item) (34). However, because of the possibility of incomplete filling in the questionnaires, 530 people entered the study. Participants fill the demographic checklist and anonymous self-administered sexual knowledge and attitudes questionnaire. Participants were asked to place the completed questionnaires into a wrapped box.

**Data analysis**

Data analysis was performed using several methods as followed: 

**Validity**

Face, content and construct validity were assessed. 

Face validity were evaluated through qualitative and quantitative methods, participants were requested to complete the questionnaire and give their viewpoint about simplicity and clarity in responding to the questionnaire. 40 premarital women and men completed the questionnaire; Participants stated that they did not have trouble with reading and understanding the items. In the quantitative approch impact score was evaluated. For this, the ratio of participants who identified the items as important and very important were calculated and impact score ≥1.5 would be accepted (35).

Content validity: A panel of experts (Who has published at least one article about sexual issues) including 15 investigators assessed the content validity of the questionnaire. Experts specialized in: reproductive and sexual health (8 people), health education and health promotion (2 people), obstetrician and gynecologist (2 people), psychiatrist (3 people). Content validity was done using qualitative and quantitative techniques. In terms of qualitative traits, grammar, wording and allocation of items were reviewed and edited based on expert opinions. In the quantitative techniques, content validity ratio (CVR) and content validity (CVI) were assessed (36). The necessity of items was evaluated using the three-point grading scale: not necessary, useful but not necessary and necessary. After evaluating the experts, CVR was calculated for each item.

The CVI based on three criteria; relevancy, simplicity, and clarity was calculated. Each item of questionnaire was evaluated in a four category likert scale from 1=not relevant, not simple and not clear to 4=very relevant, very simple and very clear. The CVI was calculated as the proportion of items that received a rating of 3 or 4 by the experts. The items were accepted only if CVI and CVR were greater than 0.79 and 0.49 respectively and otherwise refused (37). Construct validity was applied through Exploratory Factor Analysis (EFA) on 500 pre-marriage women and men. Principal component analysis with varimax rotation was performed to identify the primary construct of sexual knowledge and attitudes sections. A factor loading of ≥0.4 assumed acceptable (38). 

**Reliability**

Cronbach’s alpha coefficients were calculated to assess internal consistency. Test-retes reliability was performed to assess the stability through test-retest correlation. As such 40 participants randomly selected from the original group and completed the questionnaire twice with a 2 wk interval. Data analysis (means, standard deviations, frequencies, EFA, Cronbach's alpha and coralation between test-retest with 2 wk interval) was conducted. 

**Ethical consideration**

The ethics committee of the faculty of medicine of Tarbiat Modares University, Tehran, Iran approved the study (the approval letter was issued under the No. 1640). The participants were informed that participation was voluntary and anonymous. Informed written consent was obtained from each participant.

**Statistical analysis**

Several statistical methods were used to analyze the data: Validity was assessed through content, face, and construct validity. The Cronbach’s alpha coefficient was used to calculated the internal consistency. Test-retest reliability was conducted to assess stability of the scale. The SPSS software (Statistical Package for the Social Sciences, version 17.0, SPSS Inc, Chicago, Illinois, USA) was used for exploratory factor analysis, calculation of correlation coefficient and frequencies of baseline characteristics of the study participants.

## Results

**Participants**

In all 530 individuals were approached. Of these, 500 premarital men and women (250 men and 250 women) agreed to participate in the study and completed the questionnaire. The mean age of participants was 24.1±3.9 yr. The characteristics of the participants are presented in Table I.

**Validity**

Face validity: In the qualitative face validity, 40 participants of newlywed men and women indicated that they have had no difficulties in reading and understanding the items. Quantitative face validity examined by calculation of the impact score. Impact score of each item had ranged from 1.9-4.7 (Table II, III). Therefore, no items were omitted. Mean impact score was 3.19 and 3.22 for Sexual knowledge and sexual attitudes sections respectively.

Content validity: In the quantitative content validity, in all 12 items based on CVI and CVR less than 0.79 and 0.49 were omitted. Consequently, the number of items decreased to 37 for the sexual knowledge and to 39 for the sexual attitudes. CVR and CVI of each omitted items for both sexual knowledge and sexual attitudes sections were between -0.06 to 0.47 and 0.26-0.73 respectively. The CVR and CVI of remained items were between 0.6-1 and 0.8-1 respectively (Table II, III). 

Construct validity: The pre-final version of SKAS-PC with 37 items as sexual knowledge and 39 items as sexual attitudes was provided for the EFA. 

**Factor analysis of the sexual knowledge section**

The Kaiser-Meyer-Olkin (KMO) and Bartlett’s test confirmed that the sample size was proper for factor analysis (KMO =0.78, χ2=5234, p<0.001). In the initial step ten factors with eigenvalues >1 and factor loading≥0.4 extracted using varimax rotation. These factors explained a 60% of thevariance.

To simplify the interpretation and naming of the factors, five factors that explained 43.05% of the variance were accepted, and 4 items with a loading less than 0.4 were eliminated in the analysis of the factors. 

The extracted factors contained 33 items including factor 1 (sexual biology) with 7 items, factor 2 (sexually transmitted diseases) with 8 items, factors 3 (sexual relationship) with 6 items, factor 4 (the probability of pregnancy and its prevention) with 9 items and factor 5 (the anatomy of the genitalia) with 3 items. The results are shown in Table III. Total score of the sexual knowledge section was in the range of -33 to 33, a higher positive score indicated a higher level of sexual knowledge.

**Factor analysis of the sexual attitudes section**

The Kaiser-Meyer-Olkin (KMO) and Bartlett’s test confirmed that the sample size was proper for factor analysis ((KMO=0.83, X^2^=6094, p<0.001)1). In the initial step ten factors with eigenvalues >1 and factor loading ≥0.4 extracted using varimax rotation. These factors explained a 60. 50% of the variance.

For simplicity the explanation and naming of the factors, six factors that explained 51.0% of the variance were accepted, and 5 items with a loading less than 0.4 were eliminated. The extracted factors contained 34 items including factor 1 (sexual satisfaction) with 9 items, factor 2 (negotiation of sexual issues between spouse) with 8 items, factor 3 (the importance of sex in life) with 6 items, factor 4 (sexual concerns) with 6 items, factor 5 (initiation of sex by the woman) with 3 items and factor 6 (shared pleasure of sex) with 2 items. The findings are presented in Table II. Total score of sexual attitudes section was in the range of 34 to 170. 

**Reliability**

The internal consistency was evaluated using the Cronbach's alpha coefficient, which was 0.84 for the sexual knowledge and 0.81 for the sexual attitudes section. The Cronbach's alpha values obtained for all the subscales of the sexual knowledge section ranged from 0.66-0.85 and from 0.67-0.87 for the subscales of the sexual attitudes section. In addition, the test-retest correlation for sexual knowledge and sexual attitude sections was 0.74 and 0.82 respectively and for the subscales of two sections ranged from 0.64-0.88 and from 0.67-0.87 respectively (Table IV).

**Table I:** Baseline characteristics of the study participants (n=500

**Variables**		**No (%)**
Age		
	16-20	109 (21.8)
	21-25	203 (40.6
	>25	188 (37.6)
Gender		
	Male	250 (50)
	Female	250 (50)
Education		
	Primary	46 (9.2)
	Secondary	207 (41.4)
	higher	247 (49.4)
Male job		
	Employed	241 (96.8)
	Unemployed	9 (3.2)
Female job		
	Employed	60 (23.6)
	Unemployed	190 (76.4)
Mother education		
	Illiterate	53 (10.6)
	Primary	266 (53.2)
	Secondary	150 (30.0)
	Higher	31 (6.2)
Father education		
	Illiterate	42 (8.4)
	Primary	178 (35.6)
	Secondary	222 (44.4)
	Higher	58 (11.6)
Source of sexual information		
	Parents/Siblings/other relatives	148 (29.6)
	Classmates/ friends	112 (22.4)
	Teacher/ counselor	64 (12.8)
Health care provider		
	Books/magazines	96 (19.2)
	Internet/satellite/movie	80 (16.0)

Data are presented as n (%)

**Table II:** Results of construct validity (exploratory factor analysis), content validity (CVR and CVI) and face validity (Impact scores) of the sexual attitudes section

**Items and items number in 6 factors of sexual attitudes section** *		**Factors loading**																	**CVR**	**CVI**		**Impact score**	
		**1**		**2**			**3**			**4**			**5**				**6**						
1 Sexual satisfaction																							
	8. Couples should make sex more interesting through some initiatives	0.81		-0.05			0.09			0.03			-0.02				-0.01		0.87	0.8		3.9	
	7. Even after ejaculation, men can make the effort for their woman's orgasm.	0.79		0.01			-0.09			0.07			0.04				0.06		0.87	0.8		3.7	
	5. Foreplay before intercourse is a waste of time	0.78		0.04			-0.08			-0.01			0.11				0.08		1.0	1.0		3.6	
	3 It is not always necessary to have an orgasm to enjoy sex	0.764		0.10			-0.01			-0.03			0.11				0.04		1.0	0.87		3.4	
	2. Both spouses should consent to having sex	0.75		0.06			0.07			-0.02			0.07				0.08		0.87	0.93		4.2	
	4. Women are obligated to say yes to their husband's sexual requests under any circumstances	0.64		-0.09			-0.04			0.00			-0.018				0.062		0.87	0.93		2.5	
	1. Most women consent to sex only to keep their husband	0.64		0.05			0.07			0.01			0.07				0.06		0.87	1.0		3.3	
	6. Behaviors such as hugging and love after sexual intercourse lead to more satisfaction	0.62		0.08			0.12			-0.02			0.03				0.01		1.0	0.87		4.7	
	10. The type of sex should be agreed by both spouses.	0.40		-0.05			0.20			-0.06			-0.22				0.04		0.73	0.8		3.7	
2 Negotiation of sexual issues between spouse																							
	17. Couples should be ready to negotiate with each other about their sexual demands	-0.04		0.71				0.13			0.01			-0.00			0.04		0.87	0.8		3.9	
	14. If someone loves their spouse, they should find out their sexual needs and there is no need for a negotiation	-0.02		0.69				0.16			-0.01			-0.069			-0.03		0.73	1.0		3.5	
	16. Talking about sexual desires diminishes respect between the couples	0.06		0.65				0.16			0.10			-0.06			0.23		0.87	0.93		3.4	
	19. Women are afraid to talk about their sexual information with their spouse for fear of his suspicion	0.05		0.63				0.19			0.16			0.04			0.04		1.0	1.0		2.2	
	15. Couples should guide each other in finding sensitive parts of their body	-0.13		0.61				-0.01			0.09			0.05			-0.04		0.87	0.93		4.2	
	18. To avoid sex, it is better women don't turn to ways other than negotiation with her husband	0.10		0.61				0.36			0.01			0.02			0.046		0.87	1.0		2.6	
	13.Women should be immodest to tell their husband how to stimulate them	0.02		0.59				0.10			-0.07			0.12			-0.15		1.0	0.87		3.4	
	20. Couples can talk to each other about some of their sexual fantasies	0.14		0.58				0.20			0.11			-0.02			0.06		0.87	1.0		3.2	
3 The importance of sex in life																							
	24. The couple's attention to each other's sexual needs affects their non-sexual relationship	0.14			0.15			0.75				0.10			-0.06		-0.03		0.87		0.93	3.4	
	22. Sex becomes mundane and boring several years after marriage	-0.01			0.23			0.71				0.01			0.04		0.08		1.0		0.87	3.3	
	27. Most women consider having sex with their husband as a commitment	0.07			0.23			0.71				-0.06			-0.01		-0.01		0.86		1.0	3.3	
	26. Sex is an important part of a romantic life	0.09			0.26			0.68				-0.05			0.02		0.04		0.87		0.8	3.2	
	23. Having satisfying sex leads to a peace of mind	-0.06			0.06			0.68				0.07			0.06		-0.02		0.87		0.87	3.8	
	25. Having safe sex is an important obligation for couples	-0.01			0.12			0.65				0.17			0.06		-0.02		1.0		0.87	4.3	
4 Sexual concerns																							
	28. Having sex more than twice per week is harmful	-0.01			-0.01			0.014				0.75				-0.038		-0.03	0.87	0.8		3.3	
	32. There is a fear of sex, because it naturally may lead to hurt	0.01			0.07			-0.07				0.72				0.039		-0.04	1.0	0.93		2.1	
	30. Sexual intercourse requires a high physical strength that not everyone has it.	-0.09			-0.07			-0.01				0.71				-0.06		-0.06	0.20	o.43		2.1	
	31 Sex should be occur when couples have prior planning	-0.00			0.32			0.11				0.64				0.01		0.03	0.73	1.0		2.9	
	29. It is important that couples reach to orgasm at the same time	-0.03			0.11			0.03				0.56				0.04		0.02	0.73	0.87		3.4	
	33. Men should maintain their erection until their wife's orgasm	0.15			0.18			0.15				0.48				0.02		0.08	1.0	1.0		2.2	
5 Initiation of sex by the woman																							
	35. I have good feeling to women who reveal their sexual desires to their husband	0.01				0.00		0.01				-0.00				0.86		0.07	0.73	0.93			3.7
	34. Men do not always prefer to initiate sex themselves	0.10				0.02		-0.00				0.01				0.85		0.04	0.87	0.87			3.3
	36. When a woman initiates sex, her personality gets tarnished	0.13				0.03		0.10				-0.01				0.76		0.01	1.0	0.87			3.5
6 Shared pleasure of sex																							
	11. Each of couples in sex should think about own enjoyment		0.11		-0.03				0.03			-0.01				0.05		0.83	1.0		0.8	2.1	
	12. Sex is a two-sided relationship and couples should think about each other's enjoyment		0.18		-0.03				0.06			-0.01				0.04		0.81	0.87		0.93	2.3	

CVR: Content validity ratio

CVI: Content validity index

*: Reverse scoring items: 1, 4, 5, 11, 13, 14, 16, 19, 22, 28, 29, 30, 31, 32, 33, 36

**Table III:** Results of construct validity (exploratory factor analysis), content validity (CVR and CVI) and face validity (Impact scores) of the sexual knowledge section

**Items and items number in five factors of sexual knowledge section** *		**Factors loading**					**CVR**	**CVI**	**Impact score**
		**1**	**2**	**3**	**4**	**5**			
1 Sexual biology									
	4. The head of the penis is the most sensitive part to stimulation	0.81	0.06	0.03	0.06	0.02	0.6	0.80	2.7
	3. Following sexual stimulation, increased blood flow into the penis causes it to stiffen	0.80	0.07	0.06	0.05	0.02	0.73	0.80	2.4
	2. Direct or indirect stimulation of the clitoris is necessary for most women to reach orgasm	0.75	0.20	0.08	0.04	0.17	1.0	1.0	2.9
	6. Women require more time than men to reach orgasm	0.72	0.06	0.01	0.10	0.08	0.87	0.87	3.6
	8. The vagina becomes wet and lubricated with sexual stimulation	0.67	0.17	0.03	-0.01	0.09	0.87	0.93	3.9
	5. Men require at least a few minutes to reach a second orgasm after the first one	0.56	0.19	-0.11	-0.03	0.06	1.0	0.87	3.2
	7. Achieve the orgasm in women has a similar shape	0.53	0.33	0.03	0.15	0.01	0.60	0.80	3.3
2 Sexually transmitted diseases (STD)									
	29. Some STDs are asymptomatic	0.15	0.65	0.05	0.05	-0.01	1.0	1.0	2.3
	30. People may have several STDs at the same time	0.16	0.64	0.06	0.11	0.08	0.73	0.80	1.9
	32. STDs can have adverse consequences such as infertility	0.08	0.63	0.01	0.12	0.03	0.6	0.80	2.2
	36. The majority of STDs can also be transmitted through anal sex	0.06	0.63	-0.02	0.14	0.04	0.73	0.80	3.5
	31. The majority of STDs can also be transmitted through oral sex	0.11	0.54	0.07	0.04	0.01	1.0	0.87	3.8
	33. STDs are only transmitted through vaginal penetration	0.09	0.50	0.02	0.11	0.16	0.87	0.87	4
	34. Using condoms is an effective prevention method for most STDs, including HIV and hepatitis	-0.01	0.46	0.17	-0.02	0.16	0.73	0.87	3.1
	35. STDs symptoms may include painful urination, burning and itching	0.06	0.45	0.17	-0.04	0.22	0.87	0.93	3.4
3 Sexual relationship									
	17. Couples can have sexual fantasies during sex	0.01	0.12	0.74	0.07	0.01	0.7	0.93	3.1
	9. Reaching orgasm is necessary for both men and women every time they have sex	0.01	0.17	0.72	0.018	0.02	0.87	0.93	2.3
	15. foreplay is the most important part of a satisfying sex	-0.05	0.07	0.70	-0.01	-0.01	1	1	4
	18. Enjoy of sex can be exist for a longer time even before and after the orgasm	-0.03	0.04	0.70	0.03	-0.02	0.87	0.87	0.87
	16. Lubricants can be used during sex	0.04	0.18	0.62	0.02	0.01	0.87	0.87	4.2
	23. The natural sex position is the man to be on top	0.02	-0.14	0.46	0.21	0.15	0.6	0.80	2.3
4 The probability of pregnancy and its prevention									
	24. It is possible to get pregnant even with an intact hymen	0.11	-0.04	-0.06	0.68	0.03	0.73	0.87	2.2
	25. Pregnancy is possible even by having sex only once	0.05	0.06	-0.04	0.66	0.06	0.73	0.93	3.5
	28 Vaginal penetration is necessary for pregnancy to occur	0.04	0.09	-0.08	0.63	0.01	0.87	0.80	3.9
	22. Women can never get pregnant during menstruation cycle	-0.08	0.08	0.16	0.57	0.01	1.0	1.0	3.4
	27. The best time for a woman to get pregnant is around the middle	0.11	0.09	0.09	0.55	0.01	0.73	0.87	3.7
	26. In emergency contraceptive pills, one pill is taken for each intercourse	0.02	0.31	0.11	0.45	0.03	0.87	0.93	3.4
	14. Contraceptive pills are an effective contraceptive method	0.07	-0.11	0.25	0.45	-0.01	0.73	0.80	4
	21. For condoms to be effective, they should be used during the entire sexual intercourse	0.05	0.37	0.13	0.43	-0.09	0.87	0.93	3.3
	19. Withdrawal is an effective contraceptive method	-0.05	0.29	-0.05	0.41	0.03	0.60	0.87	3.6
5 The anatomy of the genitalia									
	12. Women's clitoris is visible from the outside	0.30	0.13	0.03	0.04	0.86	0.87	0.80	3.7
	11 The rupture of hymen is always accompanied by bleeding	0.26	0.14	0.01	0.03	0.71	0.80	1.0	3.3
	10. An erected penis is usually about 13 cm	0.12	0.10	0.01	0.01	0.61	0.60	0.87	2.9

CVR: Content validity ratio

CVI: Content validity index

STDs: Sexually transmitted diseases

HIV: Human immunodeficiency virus

*: Reverse scoring items: 7, 11, 26, 19, 22, 23, 28, and 33

**Table IV:** Reliability of the sexual knowledge and sexual attitudes sections

**Factors**	**Sexual knowledge section**		**Sexual attitudes section**	
	**Internal consistency**	**Test-retest correlation**	**Internal consistency**	**Test-retest correlation**
1	0.85	0.66	0.82	0.77
2	0.75	0.64	0.87	0.76
3	0.76	0.75	0.74	0.70
4	0.74	0.84	0.82	0.72
5	0.66	0.73	0.79	0.67
6	-	.	0.67	0.66
All	0.84	0.74	0.81	0.82

## Discussion

Sexual knowledge and sexual attitudes in couples that are going to marry is an important issue. It is argued that the defect in the sexual relationship in young men and women could lead to marital break down (39, 40). The SKAS-PC was designed and developed to assess such an important issue. We undertook a robust methodology to do so and now the findings confirm that SKAS-PC is able to measure sexual knowledge and attitudes in premarital couples. In addition, the SKAS-PC gives a total score and can be utilized when needed. Thus, it provides healthcare professionals with a means to measure sexual health knowledge and attitudes of premarital couples. As most participants completed the scale without any difficulties in approximately 20 minu, we believe that the SKAS-PC can be used easily for sexual health interventions.

There are a few questionnaires in Iran to measure sexual Knowledge and attitudes. For instance, sexual knowledge and attitudes scale contain 30 items (15 items for each subscale) was designed by Besharat and colleagues (27). Construct, convergent and discriminate validity, and reliability were assessed. The validity and reliability were found to be accurate. This questionnaire has been designed for married men and women who already have some sexual experience and have a better sexual knowledge and attitudes compared to pre-marriage couples. Furthermore, 15 items alone may not be able to assess all aspects of sexual knowledge as well as attitudes. Khajehei's and co-workers, evaluated sexual and reproduction knowledge and attitudes of the pre-marriage couples in Shiraz in 2010 through a researcher-made questionnaire (29).

The questionnaire was provided based on the materials presented in the 1 hr training session for pre-marriage couples. They confirmed the reliability of the scale by Cronbach α-coefficient, and validity by content validity. Given that these classes are not normally held for discussing sexual issues, questionnaire does not cover all aspects of sexual knowledge and attitudes. For example, their questionnaire contained only seven items about sexual attitudes that all items assessed participants' attitudes about the necessity of sexual health education classes. Several questionnaires exist to measure sexual knowledge and attitudes in other countries. For instance, the "Mathtech Sexuality Questionnaire for Adolescents" developed by Kirby (41). 

The questionnaire consists of Mathtech Knowledge and Mathtech Attitudes and Values. The sexual Knowledge areas such as physical development, social relationships and marriage might not be applicable for assessing sexual knowledge of premarital couples. The sexual attitudes areas such as understanding of emotional needs, understanding of personal social behavior, social relationship and attitudes toward premarital intercourse might not be useful for assessing sexual attitudes of Iranian premarital couples. However, some aspects of sexual knowledge and attitudes, such as the likelihood of pregnancy, contraception, the importance of sex in life were similar to the dimensions extracted in our questionnaire. A sexual knowledge questionnaire designed by Gough co-workers (42). It contained areas such as reproduction, contraception, menstruation, and menopause. The designer of the questionnaire believes that it is mostly suitable for the highly educated and the intellectual women. Monge et al developed another questionnaire in this field "Acquisition of sexual Information Test". The questionnaire contained four domains including 1) sexually transmitted diseases, 2) contraception, and sexual relationship 3) female sexual biology 4) male sexual biology (43). Although the names of their domains are similar to our domains, items are different. In this regards, Iranian researchers tested their questionnaire on women. They found the majority of participants indicated that the overall level of questions was high and some of the items were not related to sexual knowledge. Many questions gained a CVR and CVI less than acceptable rate. They concluded acquisition sexual information test seems to be culturally inappropriate (26). 

Similarly, Hendrick and colleagues designed a sexual attitudes questionnaire including some dimensions such as abortion, nudity, premarital sex, prostitution, and homosexuality (25). In addition, a new version of "The Sexual Knowledge and Attitude Test for Adolescents" developed in 2005 by Fullard and Scheier (44). It contains six subscales including premarital sexuality, rape, coercion, masturbation, abortion, homosexuality, and Pornography. We felt such items might not be of use for premarital couples as seemed very personal and difficult to answer. However, sexual knowledge and attitude are deeply affected by several factors such as biological, cultural, social, ethical, legal, historical, and religious factors (45).

**Limitations**

We only used the questionnaire in Iran and thus its validity in other settings is not guaranteed.In addition, convergent validity not evaluated. Eventually confirmatory factor analysis for the scale is suggested. 

## Conclusion

Given the lack of sexual knowledge and attitudes questionnaires for premarital couples, the findings suggest that the SKAS-PC is a valid and reliable instrument. More studies are needed to create stranger psychometric properties for the scale.

## Note

***This article extracted from Ph.D. Thesis. (Zohreh Sadat)***
